# Developing Predictive Models for Carrying Ability of Micro-Plastics towards Organic Pollutants

**DOI:** 10.3390/molecules24091784

**Published:** 2019-05-08

**Authors:** Xiaoxuan Wei, Miao Li, Yifei Wang, Lingmin Jin, Guangcai Ma, Haiying Yu

**Affiliations:** College of Geography and Environmental Sciences, Zhejiang Normal University, Yingbin Avenue 688, Jinhua 321004, China; xxwei@zjnu.edu.cn (X.W.); mli0201@163.com (M.L.); yfwang1103@126.com (Y.W.); jlm3630@163.com (L.J.); magc@zjnu.edu.cn (G.M.)

**Keywords:** microplastic, adsorption partition coefficients (log *K*_d_), predictive model, adsorption mechanism

## Abstract

Microplastics, which have been frequently detected worldwide, are strong adsorbents for organic pollutants and may alter their environmental behavior and toxicity in the environment. To completely state the risk of microplastics and their coexisting organics, the adsorption behavior of microplastics is a critical issue that needs to be clarified. Thus, the microplastic/water partition coefficient (log *K*_d_) of organics was investigated by in silico method here. Five log *K*_d_ predictive models were developed for the partition of organics in polyethylene/seawater, polyethylene/freshwater, polyethylene/pure water, polypropylene/seawater, and polystyrene/seawater. The statistical results indicate that the established models have good robustness and predictive ability. Analyzing the descriptors selected by different models finds that hydrophobic interaction is the main adsorption mechanism, and π−π interaction also plays a crucial role for the microplastics containing benzene rings. Hydrogen bond basicity and cavity formation energy of compounds can determine their partition tendency. The distinct crystallinity and aromaticity make different microplastics exhibit disparate adsorption carrying ability. Environmental medium with high salinity can enhance the adsorption of organics and microplastics by increasing their induced dipole effect. The models developed in this study can not only be used to estimate the log *K*_d_ values, but also provide some necessary mechanism information for the further risk studies of microplastics.

## 1. Introduction

Microplastics have become an emerging global environmental pollution problem [1]. They have been frequently detected in sediments [2,3], organisms [4], seawater [5], freshwater [6], and even in subpolar waters [7]. Microplastics can exist in the environment for a long time, bringing significant environmental and ecological risks. For example, microplastics can block the light transmission in water, which may affect the light absorption of various organisms. More researches proved that microplastics can be ingested by organisms, causing blockages in the digestive system, which can lead to inflammation and chronic toxicity [4,8]. In addition to the effects of microplastics themselves, they also can alter the existent form, regional concentration, environmental persistence, environmental behavior, ecological risk, etc. of the coexisting pollutants (e.g., organic pollutants) via adsorption. For example, it has been proven that microplastics can inhibit the dissipation and transformation of phenanthrene in water and enhance its bioaccumulation in *Daphnia magna* body [6]. Therefore, understanding the adsorption interaction between microplastics and organic pollutants is of great importance to clarify their environmental risk deeply and completely.

Due to the small particle size (<5 mm) and large specific surface area of microplastics, they can easily adsorb ambient organic pollutants [9,10]. Generally, the adsorption ability of microplastics towards organic pollutants can be expressed by the equilibrium coefficient of organic pollutants partitioning between microplastics and water (*K*_d_) [11]. Previous experimental studies showed that the values of *K*_d_ can be significantly influenced by the properties of microplastics, types of organic pollutants, conditions of environmental medium, and so on. For example, log *K*_d_ (L/kg) values for the adsorption of aged polystyrene (PS) microplastics in pure water ranged from 1.37 for dichloromethane to 3.9 for n-hexane [12]. For aliphatic and aromatic organic pollutants, *K*_d_ values for different microplastics increased in the order: polyamide < polyethylene (PE) < polyvinylchloride < PS [13]. The *K*_d_ value (PS) of perfluorobutanoic acid in seawater is almost 31 times that of freshwater [14]. Thus, to reveal the adsorption carrying ability of different microplastics towards huge and ever-increasing number of organic pollutants in various environmental conditions, a large number of *K*_d_ values have to be determined.

Given the research of microplastics is only in the primary stage, the reported *K*_d_ values are far from meeting the needs of further research [15]. Currently, *K*_d_ values are usually acquired via adsorption experiments. This method always needs a long equilibrium time and strict experimental control, leading to high cost and time delay. Quantitative structure–property relationship (QSPR) models can just make up for these shortcomings. Mechanism-based QSPR models, such as polyparameter linear free energy relationship (pp-LFER) model, can not only provide predictive *K*_d_ values efficiently, but also promote the adsorption mechanism analysis [16,17,18]. Currently, few *K*_d_ predictive models for microplastics have been reported. However, most of these models were developed only based on the experimental *K*_d_ values obtained in the corresponding studies, and used to analyze the contribution of individual molecular interactions to overall sorption [12,13]. Therefore, it is still necessary to develop *K*_d_ predictive models based on QSPR for estimating the adsorption carrying ability of different microplastics towards organic pollutants in various waters, and preliminarily discussing the adsorption mechanisms.

In this study, we collected *K*_d_ values for the three most frequently detected microplastics, including PE, polypropylene (PP), and PS [19]. Our main purposes were (1) to establish models for predicting *K*_d_ values of polychlorinated biphenyls, chlorobenzenes, polycyclic aromatic hydrocarbons, antibiotics, aromatic hydrocarbons, aliphatic hydrocarbons, hexachlorocyclohexanes, and perfluorinated compounds for PE, PP, and PS in seawater, freshwater, and pure water; (2) to explore the adsorption interaction mechanism; (3) to discuss the effects of microplastic type and environmental condition on the *K*_d_ values. 

## 2. Results and Discussion

### 2.1. Predictive Models for the Adsorption Ability of PE

The pp-LFER models of log *K*_d_ were developed for the partition of organic pollutants between PE and three types of water (seawater, freshwater, and pure water):

log *K*_d_ = (−3.822 ± 0.222) × *B* + (3.054 ± 0.348) × *V* + (1.293 ± 0.173) × *E* + (−1.410 ± 0.684) (1)

log *K*_d_ = (−3.302 ± 0.233) × *B* +(5.594 ± 0.887) × *V* + (−3.960 ± 1.691) (2)

log *K*_d_ = (−2.562 ± 0. 117) × *B* +(2.895 ± 0.140) × *V* + (0.902 ± 0.228) (3)

where, *B* represents for the hydrogen bond accepting ability (basicity), *V* is the McGowan’s molar volume and *E* refers to the excess molar refraction. Models (1)–(3) correspond to the adsorption in seawater, freshwater, and pure water, respectively. As shown in Williams plot for Model (3) (Figure S1 in the Supplementary Materials, SM), the absolute standardized predictive residuals (*SR*) value of 2,2′,4,5′,6-pentachlorobiphenyl (3.439) is larger than 3. Thus, it is diagnosed as an outlier. After removing it, Model (4) was yielded for the adsorption of PE in pure water:

log *K*_d_ = (−2.594 ± 0.101) × *B* + (2.940 ± 0.121) × *V* + (0.864 ± 0.195) (4)

**Figure 1:**
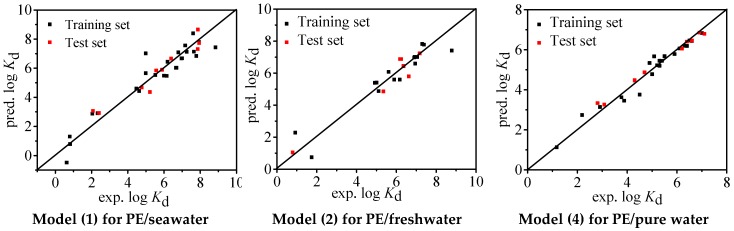
Fitting plots of experimental and predicted log *K*_d_ by Models (1), (2), and (4). PE, polyethelyne.

**Figure 2:**
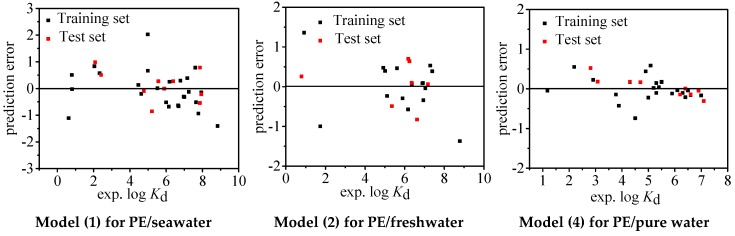
Distributions of prediction errors of log *K*_d_ calculated by Models (1), (2), and (4). PE, polyethelyne.

The statistical parameters of Models (1), (2), and (4) are shown in Table 1. For all the three models, *R*^2^ = 0.911, 0.909, and 0.978; *Q*^2^ = 0.911, 0.909, and 0.978; and *RMSE* = 0.677, 0.608, and 0.222, respectively, suggesting significant goodness of fit statistics and the combination of molecular descriptors can explain 91%, 91%, and 98% variability of log *K*_d_ for the whole dataset, respectively. As shown in Table S1, all the *VIF* values (1.065~1.471) are < 10, indicating nonexistence of multicollinearity for the present models. The fitting plots (Figure 1) illustrate a favorable consistence between the experimental and predicted log *K*_d_ values. The pattern of predictive errors shown in Figure 2 reveals that there is no dependence on experimental log *K*_d_ values and consequently no systematic error for the models, which is also verified by *BIAS* = 0.000 (Table 1).

The statistical parameters of simulated external validation were listed in Table 1. Comparing with the models developed by the whole dataset, redeveloped pp-LFER models (S1–S3) based on 70% experimental data and descriptors in Models (1), (2), and (4) show similar regression performance (including *R*^2^, *Q*^2^, *RMSE*, and *MAE*) and regression coefficients. The results prove that the models are statistically stable and there is no casual correlation, as the training subsets are randomly assigned. The predictive performance of each rebuilt model to the corresponding test set (30% subset, shown by the superscript of b in Table 2) was listed in Table 1, *Q*^2^ = 0.923–0.977, *RMSE* = 0.251–0.583, and *MAE* = 0.188–0.453, indicating very good predictive quality of the developed pp-LFERs. Moreover, the results of leave-one-out cross validation (*Q*^2^_CV_ = 0.911–0.917) also reveal a good degree of robustness and internal predictive goodness [20].

**Figure 3:**
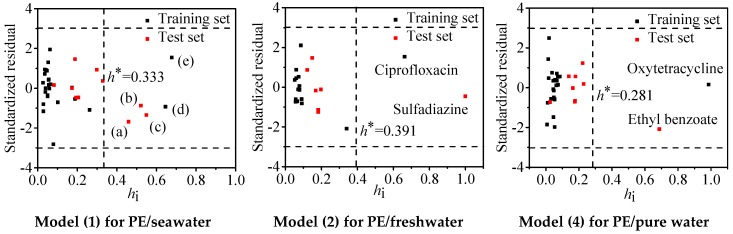
Williams plots for the applicability domain of Models (1), (2), and (4). The *h_i_* refers to the verse leverage value. (a): δ-hexachlorocyclohexane, (b): α-hexachlorocyclohexane, (c): pentachlorobenzene, (d): dioctyl phthalate, (e): oxytetracycline.

Williams plots were applied to determine the application domain of the regression Models (1), (2), and (4). The alert value h^*^ were calculated to be 0.333, 0.391, and 0.281, respectively. As shown in Figure 3, there are five (δ-hexachlorocyclohexane, α-hexachlorocyclohexane, pentachlorobenzene, dioctyl phthalate, and oxytetracycline), two (ciprofloxacin and sulfadiazine), and two (ethyl benzoate and oxytetracycline) compounds located at the right side of h^*^ for Models (1), (2), and (4), respectively. However, they are not diagnosed to be outliers as their absolute *SR* values are <3. This phenomenon proves the developed models have excellent generalization capabilities in their descriptor matrix. It follows that Models (1), (2), and (4) can be used to predict log *K*_d_ values for the adsorption of organics that have similar structures with the chemicals in Table 2 towards PE in seawater, freshwater, and pure water, respectively.

For all the three log *K*_d_ predictive models for PE in seawater, freshwater, and pure water, hydrogen bond basicity (*B*) and McGowan’s molar volume (*V*) were selected. The experimental log *K*_d_ values most significantly correlate with *B*, which yields negative correlation coefficients of *B* (−3.822, −3.302, and −2.594) in Models (1), (2), and (4), indicating that the hydrogen bond basicity of organics plays an important inhibition role in the adsorption of PE. This is because compounds with high hydrogen bond basicity can easily act as H-bond receptors to form H-Bond with the H atoms in water molecules. Thus, these organics prefer to dissolve in water rather than be absorbed on the surface of PE. For example, the structure analysis of compounds listed in Table 2 (structures are shown in Table S2) found that the compounds (such as dioctyl phthalate, oxytetracycline, trimethoprim, etc.) containing O atoms in the structure have larger *B* values and smaller log *K*_d_ values. 

*V* can characterize the cavity formation energy and describe the dispersion and hydrophobic interactions. As water is a highly organized and very cohesive solvent, a large *V* value indicates the compound needs high cavity formation energy to dissolve in water [21]. Thus, the organics with large *V* values prefer partitioning into the particulate phase and consequently result in large log *K*_d_ values. 

Especially for the adsorption of PE in seawater, one more descriptor, excess molar refraction (*E*), was selected. *E* is a term accounting for the induction effects (i.e., π and n-electron pair interactions). The higher *E* value, the stronger induced dipole interaction occurs between organics and PE. Thus, these organic compounds tend to be absorbed by PE. Moreover, the high salinity of seawater can significantly enhance the induced dipole interaction. As a result, *E* value plays a more important role in determining the *K*_d_ values of chemicals between PE and seawater than freshwater and pure water. As shown in Table 2, log *K*_d_ values in seawater are basically larger than that in freshwater and pure water. In brief, the distribution behavior of the studied organics between PE and water is mainly affected by the hydrogen bond basicity and cavity formation effect. Thus, it is inferred that hydrophobic interaction is an important absorption mechanism. For the adsorption in seawater, induced dipole effect is another important driving force.

### 2.2. Predictive Model for the Adsorption Ability of PP in Seawater

Log *K*_d_ predictive model for the adsorption of PP in seawater was developed via pp-LFER:

log *K*_d_ = (−3.357 ± 0.219) × *B* + (1.299 ± 0.127) × *E* + (3.108 ± 0.287) (5)

Statistical parameters of *R*^2^, *Q*^2^, and *RMSE* are 0.956, 0.956, and 0.322, respectively, indicating that Model (5) has significant goodness of fit statistics and it can explain 96% variability for the whole dataset. As the *VIF* values for both descriptors are 1.001 (Table S1), the present model has no multicollinearity. As shown in Figures S2 and S3, good consistence between the experimental and predicted log *K*_d_ values and independence of predictive errors on experimental log *K*_d_ values was observed.

The simulated external validation shows that the regression coefficients (*R*^2^ = 0.914, *RMSE* = 0.471, and *MAE* = 0.371) and statistical parameters of the training subset are similar to that of the whole dataset (Table 1 and Model S4). Thus, Model (5) is statistically stable and there is no casual correlation. As shown in Table 1, the predictive performance of the new model (*Q*^2^ = 0.896, *RMSE* = 0.463, and *MAE* = 0.378) to the test subset proves a high prediction quality of the developed pp-LFER model. Moreover, *Q*^2^_CV_ value of the leave-one-out cross validation is 0.925, indicating Model (5) has good robustness and internal predictive ability. The application domain determination based on Williams plot (Figure S4) shows that there are five compounds (β-hexachlorocyclohexane, γ-hexachlorocyclohexane, sulfadiazine, trimethoprim, and benzoapyrene) located at the right side of h^*^ (0.257). While, these five compounds yield absolute *SR* values < 3, indicating they are not outliers, which further represents the excellent generalization capability of Model (5) on such chemicals. Thus, Model (5) is applicable to predict the log *K*_d_ values of PE towards the organics with similar structures with the chemicals in Table 2 in seawater.

As indicated by Model (5), the hydrogen bond basicity (*B*) and induced dipole effect (*E*) of organic compounds also play determining roles for the adsorption carrying ability of PP in seawater. However, unlike the log *K*_d_ prediction model of PE in seawater, the McGowan’s molar volume (*V*) representing the cavity formation energy is not selected in the regression model. As the addition of methyl groups in the PP structure can reduce the distance between the polymer chains and increase the crystallinity of the microplastics [22], the difference in cavity formation energy required for organics to be partitioned in seawater phase and PP phase may be reduced compared to PE, consequently resulting in a negligible contribution of *V* in the adsorption of compounds towards PP and lower log *K*_d_ values (Table 2).

### 2.3. Predictive Model for the Adsorption Ability of PS in Seawater

For the adsorption of PS in seawater, there are only 14 compounds that have available Abraham descriptor values. Basing on the experimental log *K*_d_ values of these chemicals, a pp-LFER model wad developed:

log *K*_d_ = (−14.645 ± 2.109) × *A* + (6.165 ± 0.249) (6)

where, *A* represents for the hydrogen bond donating ability (acidity). As the statistical parameters of *R*^2^ = 0.990, *Q*^2^ = 0.990, and *RMSE* = 0.168, Model (6) seems to have a significant goodness of fit statistics. However, the analysis for *A*, which is the only descriptor selected by Model (6), shows that most of the *A* value is 0. Obviously, the high regression performance of this model is spurious. Model (6) is unavailable for the log *K*_d_ prediction. Thus, a new predictive model was established with the octanol–water partition coefficient (log *K*_ow_) [13] and seven quantum chemical descriptors [23]. For the development of new model, all the collected experimental log *K*_d_ values for 28 compounds were used. The following model was yielded:

log *K*_d_ = (4.141 ± 0.371) × *π* + (0.435 ± 0.070) × log *K*_ow_ + (−3.050 ± 0.585) (7)

where, *π* is a unitless quantity which can be calculated by dividing the polarizability by molecular volume. As shown in Table 1 and Table S1, the obtained statistical parameters can prove the good regression performance and nonexistence of multicollinearity for Model (7). Meanwhile, the favorable consistence between the experimental and predicted log *K*_d_ values was observed in Figure S5. The pattern of predictive errors shown in Figure S6 reveals no systematic error for Model (7), which is also verified by *BIAS* = 0.000 (Table 1).

For the simulated external validation, similar regression coefficients and statistical parameters of the redeveloped Model S5 based on the training subset (70%) and a comparable statistical result for the test set (Table 1) were received. Moreover, *Q*^2^_CV_ value (0.906) of the leave-one-out cross validation can match the acceptable criteria well. All the results demonstrate that Model (7) has high goodness of robustness and internal predictive ability. As exhibited in the Williams plot (Figure S7), four compounds (perfluoro-1-octanesulfonyl fluoride, trimethoprim, benzoapyrene, and perfluorotetradecanoic acid) with |*SR*| < 3 locate at the right side of h^*^ (0.321), indicating that they are not outliers and that Model (7) has excellent generalization capability for them. In consequence, Model (7) is suitable for predicting the adsorption carrying ability (log *K*_d_) of PS for organic pollutants within the application domain in seawater.

Model (7) selected two molecular descriptors, including dipolarity/polarizability (*π*) and octanol–water partition coefficient (log *K*_ow_). The experimental log *K*_d_ values most significantly correlate with *π* (*R* = 0.803), which yields a positive coefficient (4.141) in the regression model. The comprehensive analysis of *π* values (Table 2) and molecular structures (Table S2) showed that the compound containing more benzene rings in the structure and stronger electron conjugation has a larger *π* value. It is inferred that the organics with large aromaticity prefer partitioning into the PS phase and consequently result in large log *K*_d_ values. This is because the introduction of phenyl groups in the PS structure allows the π−π interaction to enhance the adsorption between organics and PS in seawater. Compared to the adsorption of PE and PP in seawater, the π−π interactions between the benzene rings of PS and organic compounds make PS exhibit a higher log *K*_d_ value for most organics (Table 2). The second significant molecular descriptor is log *K*_ow_, which represents the hydrophobicity of organic compounds. The positive correlation coefficient of log *K*_ow_ (0.435) in Model (7) means that hydrophobic interaction can enhance the adsorption of organics on PS in seawater. PS containing no polar groups in the structure is strongly hydrophobic. Thus, hydrophobic interactions can be inferred to occur between PS and the hydrophobic groups of organics. This is consistent with the results of the prediction model established by Hüffer et al. based on the log *K*_ow_ values of the seven organic compounds [13].

## 3. Materials and Methods 

### 3.1. Experimental K_d_ Values

The experimental equilibrium coefficients of organic pollutants partitioning between microplastics and water (*K*_d_) were collected for PE, PP, and PS. Totally, the *K*_d_ values for 36 organic pollutants partitioning between PE and seawater, 23 organic pollutants partitioning between PE and freshwater, 33 organic pollutants partitioning between PE and pure water, 35 organic pollutants partitioning between PP and seawater, and 28 organic pollutants partitioning between PS and seawater were selected and listed in Table 2. The unit of all the *K*_d_ values was unified to L/kg, and then *K*_d_ was converted to its logarithmic forms (log *K*_d_). The experimental conditions for these *K*_d_ values are shown in Table S3. The molecular structures of all organic chemicals are shown in Table S2, including polychlorinated biphenyls, chlorobenzenes, polycyclic aromatic hydrocarbons, antibiotics, aromatic hydrocarbons, aliphatic hydrocarbons, hexachlorocyclohexanes, and perfluorinated compounds.

### 3.2. Molecular Structural Parameters

Polyparameter linear free energy relationships (pp-LFERs) can well predict the partition coefficients between two condensed phases with five Abraham descriptors, including *E*, *S*, *A*, *B*, and *V* [32,33]. Here, *E* refers to excess molar refraction; *S* stands for dipolarity/polarizability parameter; *A* represents hydrogen bond donating ability (acidity); *B* represents hydrogen bond accepting ability (basicity); and *V* is McGowan’s molar volume with units of cubic centimeters per mole/100. Values of all the pp-LFER descriptors used in this study are from UFZ-LSER database (http://www.ufz.de/lserd). The average values of each descriptor are listed in Table S4. 

For some special cases where pp-LFER model is impracticable, octanol–water partition coefficient (log *K*_ow_) and seven quantum chemical descriptors were calculated for developing available predictive model (Table S5). The selected quantum chemical descriptors include molecular mass (*M*_w_), molecular volume (*v*’), the most positive atomic charge on a hydrogen atom (*q*H^+^), the most negative net charge on an atom (*q*^−^), the ratio of average molecular polarizability and molecular volume (*π* = *α*/*v*’), covalent acidity (*ε*_α_ = *E*_LUMO_ − *E*_HOMO-water_), and covalent basicity (*ε*_β_ = *E*_LUMO-water_ − *E*_HOMO_) where *E*_HOMO_ refers to the highest occupied molecular orbital energy and *E*_LUMO_ stands for the lowest unoccupied molecular orbital energy. The log *K*_ow_ values were calculated by the EPI Suite software [34]. Quantum chemical descriptors, including *E*_HOMO_, *E*_LUMO_, *M*_w_, *v*, *α*, *q*H^+^, and *q*^−^ were extracted from the Gaussian output files. All the molecules were optimized at B3LYP/6-31G(d,p) level using Gaussian 09 program package [35]. All the optimized structures were confirmed to be local minima by vibrational frequency analyses.

### 3.3. Model Development and Validation 

The frequently used pp-LFERs established by Abraham et al. [36,37] are as follows:

log *K*_d_ = *eE* + *sS* + *aA* + *bB* + *vV* + *c* (8)

where *e*, *s*, *a*, *b*, and *v* are fitting coefficients, and *c* is a regression constant. Multiple linear regression (MLR) [38] with a step-wise algorithm embedded in soft package SPSS 21.0 was applied for variable filtration and model development. In order to characterize the fitting performance and predictive ability of the developed pp-LFERs, squared correlation coefficient (*R*^2^) and predictive squared correlation coefficient (*Q*^2^) were calculated as described in the previous article [39]. Root-mean-square error (*RMSE*) was also calculated to further assess the statistical performance of the established pp-LFER models. Variance inflating factor (*VIF*) was computed to estimate the collinearity of parameters. Calculation details for all statistical parameters are listed in the Text S1.

Simulated external validation and leave-one-out cross validation were performed to estimate the statistical robustness and predictive power. For the simulated external validation, the data set was randomly divided into a 70% training set and a 30% test subset (shown in Table 2). The training set was used to rebuild a model with the same descriptors selected by the whole dataset. Then, the log *K*_d_ values of compounds in the test subset were predicted and evaluated by the rebuilt model. Values of *R*^2^, *Q*^2^, and *RMSE* of the validation were computed to clarify the model performance. Cross validation was performed with Weka 3.8.0 [40] (University of Waikato, Hamilton, New Zealand). The cross-validated correlation coefficients (*Q*^2^_CV_) were calculated to quantify the model robustness.

For the predictive model developed with log *K*_ow_ and quantum chemical descriptors, the same establishment and validation strategies were used.

### 3.4. Outliers and Application Domain

Williams plot with the leverage value (*h*_i_) as horizontal coordinate and standardized predictive residuals (*SR*) as vertical coordinate, was introduced to visualize the application domain and determine the outliers as introduced in previous work [41]. The *h*_i_ values were computed by the Hat-matrix [42]. The compounds with absolute values of *SR* larger than 3 were designated as outliers and should be removed. Warning value (h^*^) is defined as h^*^ = 3*p*/*n* [42], in which *p* and *n* is the number of descriptors and compounds in the model, respectively. If *h*_i_ > h^*^, the compound is far away from the descriptor-matrix center. So, the Williams plot also describes the distribution of chemicals in the whole descriptor matrix.

## 4. Conclusions

Clarifying the adsorption ability and mechanism of organic pollutants on microplastics in different aqueous environments is essential for the comprehensive risk assessment of microplastics. In this study, predictive models were developed for estimating the adsorption carrying ability of PE in seawater, freshwater, and pure water, PP in seawater, and PS in seawater. The performance of each model was assessed by different validation strategies and the application domains were defined by Williams plots. Mechanism analysis found that the hydrogen bond basicity and log *K*_ow_, both of which can describe the hydrophobicity of compounds, play important roles in the adsorption, indicating that hydrophobic interaction is one of the main adsorption mechanisms. For the microplastics with benzene rings in structure, π−π interaction is also a key driving force. Besides, both the crystallinity and aromaticity of microplastics and salinity of aqueous environment can affect the carrying ability of microplastics towards organic pollutants in various waters. Actually, the adsorption between microplastics and organic pollutants is quite intricate in natural waters. This study did some preliminary explorations, and it is expected to provide efficient prediction methods for estimating the adsorption intensity and mechanism explanations for further research on the behavior and risks of microplastics.

## Figures and Tables

**Table 1:** Statistical parameters of the regression models and simulated external validation.

	*N*	*R* ^2^	*Q* ^2^	*RMSE*	*BIAS*	*MAE*	*MPE*	*MNE*
**Model (1)**	36	0.911	0.911	0.677	0.000	0.516	2.030	−1.407
**Training Set**	26	0.907	0.907	0.721	−0.043	0.541	2.030	−1.407
**Test Set**	10	0.928	0.923	0.583	0.110	0.453	0.982	−0.854
**Model (2)**	23	0.909	0.909	0.608	0.000	0.450	1.269	−1.453
**Training Set**	16	0.897	0.897	0.651	0.000	0.482	1.360	−1.371
**Test Set**	7	0.934	0.932	0.563	0.062	0.439	0.699	−0.827
**Model (3)**	33	0.963	0.963	0.280	0.000	0.203	0.962	−0.469
**Model (4)**	32	0.978	0.978	0.222	0.000	0.171	0.588	−0.462
**Training Set**	23	0.958	0.958	0.297	0.000	0.220	0.587	−0.741
**Test Set**	9	0.994	0.977	0.251	0.046	0.188	0.523	−0.309
**Model (5)**	35	0.956	0.956	0.322	0.000	0.237	0.661	−0.757
**Training Set**	25	0.914	0.914	0.471	0.000	0.371	0.904	−0.880
**Test Set**	10	0.937	0.896	0.463	0.114	0.378	0.796	−0.601
**Model (6)**	14	0.990	0.990	0.168	0.000	0.115	0.404	−0.268
**Model (7)**	28	0.933	0.933	0.507	0.000	0.363	1.471	−0.991
**Training Set**	20	0.880	0.880	0.655	0.000	0.464	0.802	−1.387
**Test Set**	8	0.832	0.812	0.981	0.250	0.840	1.3363	−1.152

**Table 2:** Experimental and predicted log *K*_d_ values of organic compounds and the values of selected molecular descriptors in Models (1), (2), (4), (5), and (7).

No.	Organic Compounds	log *K*_d_^a^		*B*	*V*	*E*	*π*	log *K*_ow_	Ref.
		Exp.	Pred.						
For the adsorption of PE in seawater, Model (1)									
1	2,4,4′-trichlorobiphenyl	6.150	5.470	0.129	1.670	1.758			[11]
2	2,4′,5-trichlorobiphenyl	6.000	5.481	0.132	1.674	1.766			[11]
3	2,2′,3,5′-tetrachlorobiphenyl ^b^	5.890	5.885	0.150	1.770	1.905			[11]
4	2,2′,5,5′-tetrachlorobiphenyl	5.900	5.894	0.147	1.770	1.903			[11]
5	2,4,4′,5-tetrachlorobiphenyl	6.660	6.026	0.130	1.792	1.903			[11]
6	2,3′,4,4′-tetrachlorobiphenyl	6.690	6.026	0.130	1.792	1.903			[11]
7	2,2′,4,5′,6-pentachlorobiphenyl	6.190	6.442	0.130	1.871	2.038			[11]
8	2,3,3′,4,4′-pentachlorobiphenyl	6.970	6.670	0.110	1.922	2.035			[11]
9	2,3′,4,4′,5-pentachlorobiphenyl	7.000	6.681	0.110	1.919	2.050			[11]
10	3,3′,4,4′,5-pentachlorobiphenyl	7.780	6.841	0.090	1.936	2.075			[11]
11	3,3′,4,4′,5,5′-hexachlorobiphenyl	8.840	7.433	0.070	2.059	2.183			[11]
12	2,2′,3,4,5,6′-hexachlorobiphenyl	6.790	7.085	0.110	1.993	2.188			[11]
13	2,2′,3,4,4′,5′-hexachlorobiphenyl	7.250	7.128	0.110	2.009	2.183			[11]
14	2,2′,4,4′,5,5′-hexachlorobiphenyl	7.650	7.134	0.113	2.015	2.183			[11]
15	2,3,3′,4,4′,5-hexachlorobiphenyl ^b^	7.860	7.318	0.090	2.041	2.196			[11]
16	2,2′,3,3′,4,4′,5-heptachlorobiphenyl	7.940	7.792	0.090	2.138	2.333			[11]
17	2,2′,3,4,4′,5,5′-heptachlorobiphenyl ^b^	7.940	7.725	0.090	2.131	2.298			[11]
18	Dichlorodiphenyltrichloroethane	4.986	7.016	0.180	2.218	1.810			[24]
19	Pentachlorobenzene ^b^	5.220	4.365	0.000	1.328	1.330			[25]
20	Hexachlorobenzene	4.630	4.431	0.130	1.451	1.475			[25]
21	Phenanthrene	4.470	4.604	0.276	1.454	2.033			[25]
22	Fluoranthene	5.520	5.530	0.247	1.585	2.354			[25]
23	Anthracene ^b^	4.770	4.676	0.272	1.454	2.077			[25]
24	Pyrene ^b^	5.570	5.841	0.282	1.585	2.698			[25]
25	Chrysene ^b^	6.390	6.661	0.325	1.823	2.897			[25]
26	Benzoapyrene	7.170	7.559	0.417	1.954	3.554			[25]
27	Dibenzanthracene ^b^	7.870	8.654	0.462	2.192	3.972			[25]
28	Benzo[g,h,i]perylene	7.610	8.392	0.455	2.084	4.004			[25]
29	Dioctyl phthalate	4.993	5.659	1.088	3.401	0.650			[24]
30	Trimethoprim	0.811	0.786	1.832	2.181	1.962			[26]
31	Sulfadiazine	0.797	1.305	1.370	1.723	2.080			[26]
32	Oxytetracycline	0.623	-0.487	3.500	3.158	3.600			[27]
33	α-Hexachlorocyclohexane ^b^	2.410	2.920	0.620	1.580	1.450			[25]
34	β-Hexachlorocyclohexane	2.040	2.875	0.632	1.580	1.450			[25]
35	γ-Hexachlorocyclohexane	2.330	2.905	0.624	1.580	1.450			[25]
36	δ-Hexachlorocyclohexane ^b^	2.080	3.062	0.583	1.580	1.450			[25]
For the adsorption of PE in freshwater, Model (2)									
37	2,4,4′-trichlorobiphenyl ^b^	5.350	4.956	0.129	1.670				[11]
38	2,4′,5-trichlorobiphenyl	5.110	4.969	0.132	1.674				[11]
39	2,2′,3,5′-tetrachlorobiphenyl	4.920	5.446	0.150	1.770				[11]
40	2,2′,5,5′-tetrachlorobiphenyl	5.010	5.456	0.147	1.770				[11]
41	2,4,4′,5-tetrachlorobiphenyl	5.890	5.635	0.130	1.792				[11]
42	2,3′,4,4′-tetrachlorobiphenyl	6.170	5.635	0.130	1.792				[11]
43	3,3′,4,4′-tetrachlorobiphenyl ^b^	6.620	5.825	0.110	1.814				[28]
44	2,2′,4,5,6′-pentachlorobiphenyl	5.610	6.077	0.130	1.871				[11]
45	2,3,3′,4,4′-pentachlorobiphenyl ^b^	6.350	6.429	0.110	1.922				[11]
46	2,3′,4,4′,5-pentachlorobiphenyl	6.360	6.412	0.110	1.919				[11]
47	3,3′,4,4′,5-pentachlorobiphenyl	6.940	6.573	0.090	1.936				[11]
48	2,2′,3,4′,5,6-hexachlorobiphenyl ^b^	6.180	6.826	0.110	1.993				[11]
49	2,2′,3,4,4′,5′-hexachlorobiphenyl	6.890	6.915	0.110	2.009				[11]
50	2,2′,4,4′,5,5′-hexachlorobiphenyl	7.040	6.939	0.113	2.015				[11]
51	2,3,3′,4,4′,5-hexachlorobiphenyl ^b^	7.170	7.160	0.090	2.041				[11]
52	3,3′,4,4′,5,5′-hexachlorobiphenyl	8.780	7.327	0.070	2.059				[11]
53	2,2′,3,4,4′,5-hexachlorobiphenyl	6.920	6.949	0.110	2.015				[28]
54	2,2′,3,4′,5′,6-hexachlorobiphenyl ^b^	6.240	6.826	0.110	1.993				[28]
55	2,2′,3,3′,4,4′,5-heptachlorobiphenyl	7.290	7.703	0.090	2.138				[11]
56	2,2′,3,4,4′,5,5′-heptachlorobiphenyl	7.390	7.664	0.090	2.131				[11]
57	Ciprofloxacin	1.741	0.614	2.520	2.305				[26]
58	Trimethoprim	0.923	2.192	1.832	2.181				[26]
59	Sulfadiazine ^b^	0.792	1.155	1.370	1.723				[26]
For the adsorption of PE in pure water, Model (4)									
60	2,2′,5-trichlorobiphenyl	4.900	5.329	0.145	1.648				[29]
61	2,4,4′-trichlorobiphenyl	5.400	5.460	0.129	1.670				[29]
62	2,4′,5-trichlorobiphenyl	5.301	5.442	0.132	1.674				[30]
63	2,2′,4,4′-tetrachlorobiphenyl	5.083	5.671	0.150	1.770				[30]
64	2,2′,5,5′-tetrachlorobiphenyl	5.500	5.701	0.147	1.770				[29]
65	2,2′,3,5-tetrachlorobiphenyl	5.500	5.671	0.150	1.770				[29]
66	2,3′,4,4′-tetrachlorobiphenyl	5.900	5.779	0.130	1.792				[29]
67	2,2′,4,5,5′-pentachlorobiphenyl ^b^	6.200	6.124	0.133	1.893				[29]
68	2,3,3′,4′,6-pentachlorobiphenyl	6.100	6.082	0.130	1.893				[29]
69	2,3′,4,4′,5-pentachlorobiphenyl	6.400	6.206	0.110	1.919				[29]
70	2,3,3′,4,4′-pentachlorobiphenyl	6.300	6.221	0.110	1.922				[29]
71	2,2′,4,4′,5,5′-hexachlorobiphenyl ^b^	6.400	6.507	0.132	2.015				[29]
72	2,2′,3,4,4′,5′-hexachlorobiphenyl	6.600	6.452	0.110	2.009				[29]
73	2,2′,3,3′,4,5-hexachlorobiphenyl ^b^	6.600	6.488	0.110	2.015				[29]
74	2,2′,3,3′,4,4′-hexachlorobiphenyl	6.500	6.488	0.110	2.015				[29]
75	2,2′,3,4′,5,5′,6-heptachlorobiphenyl ^b^	7.100	6.847	0.090	2.116				[29]
76	2,2′,3,4,4′,5,5′-heptachlorobiphenyl	7.000	6.859	0.090	2.131				[29]
77	2,2′,3,3′,4,4′,5-heptachlorobiphenyl ^b^	6.900	6.899	0.090	2.138				[29]
78	Chlorobenzene ^b^	3.080	2.920	0.070	0.839				[13]
79	Benzene	2.190	2.391	0.140	0.716				[13]
80	Toluene	2.910	2.960	0.140	0.857				[13]
81	Ethyl benzoate ^b^	2.810	3.253	0.070	0.839				[13]
82	Naphthalene	3.770	3.308	0.199	1.085				[13]
83	2-Methylanthracene	5.000	4.704	0.310	1.595				[29]
84	1-methylphenanthrene ^b^	4.700	4.848	0.275	1.595				[29]
85	9,10-Dimethylanthracene	5.300	5.343	0.300	1.736				[29]
86	3,6-dimethylphenanthrene	5.200	5.346	0.290	1.736				[29]
87	Phenanthrene	4.300	4.219	0.276	1.454				[29]
88	Anthracene ^b^	4.300	4.188	0.272	1.454				[29]
89	Oxytetracycline	1.176	1.116	3.500	3.158				[27]
90	Cyclohexane	3.880	3.716	0.000	0.845				[13]
91	Hexane	4.500	4.262	0.000	0.954				[13]
For the adsorption of polypropylene (PP) in seawater, Model (5)									
92	2,3-dichlorobiphenyl	4.980	4.450	0.163		1.628			[31]
93	2,4′-dichlorobiphenyl	4.980	4.441	0.166		1.620			[31]
94	2,4,4′-trichlorobiohenyl	5.090	4.904	0.129		1.758			[31]
95	2,2′,5,5′-tetrachlorobiphenyl	5.090	5.152	0.147		1.903			[31]
96	2,2′,3,5′-tetrachlorobiphenyl	5.140	5.143	0.150		1.905			[31]
97	3,3′,4,4′-tetrachlorobiphenyl	5.630	5.368	0.110		1.915			[31]
98	2,3′,4,4-tetrachlorobiphenyl ^b^	5.260	5.249	0.130		1.903			[31]
99	2,3′,4,4′,5-pentachlorobiphenyl	5.710	5.677	0.110		2.050			[31]
100	2,3,3′,4,4′-pentachlorobiphenyl	5.770	5.669	0.110		2.035			[31]
101	2,2′,3,4′,5-pentachlorobiphenyl ^b^	5.510	5.558	0.130		2.045			[31]
102	2,2′,3,5′,6-pentachlorobiphenyl	5.260	5.520	0.130		2.045			[31]
103	2,3,3′,4′,6-pentachlorobiphenyl	5.630	5.558	0.130		2.045			[31]
104	2,2′,4,5,5′-pentachlorobiphenyl	5.510	5.546	0.133		2.043			[31]
105	2,2′,3,3′,4,6′-hexachlorobiphenyl ^b^	6.190	5.935	0.110		2.188			[31]
106	2,3,3′,4,5,6-hexachlorobiphenyl ^b^	6.060	5.979	0.110		2.193			[31]
107	2,2′,4,4′,5,5′-hexachlorobiphenyl	6.190	5.893	0.132		2.183			[31]
108	2,2′,3,4,4′,5-hexachlorobiphenyl	5.770	5.971	0.110		2.185			[31]
109	2,2′,3,3′,4,4′-hexachlorobiphenyl	5.450	5.971	0.110		2.185			[31]
110	2,2′,3,4′,5,5′,6-heptachlorobiphenyl ^b^	5.730	6.360	0.090		2.338			[31]
111	Pentachlorobenzene ^b^	4.500	4.352	0.000		1.330			[25]
112	Hexachlorobenzene	5.010	4.253	0.130		1.475			[25]
113	Phenanthrene	4.000	4.275	0.276		2.033			[25]
114	Fluoranthene ^b^	4.790	4.904	0.247		2.354			[25]
115	Anthracene	4.290	4.330	0.272		2.077			[25]
116	Pyrene	4.800	5.104	0.282		2.698			[25]
117	Chrysene	5.510	5.557	0.325		2.897			[25]
118	Benzoapyrene ^b^	6.100	6.082	0.417		3.554			[25]
119	Dibenzanthracene	7.000	6.733	0.462		3.972			[25]
120	Benzo[g,h,i]perylene	6.690	6.598	0.455		4.004			[25]
121	Trimethoprim	0.594	0.104	1.832		1.962			[26]
122	Sulfadiazine	0.853	1.010	1.370		2.080			[26]
123	α-Hexachlorocyclohexane	2.690	2.763	0.620		1.450			[25]
124	β-Hexachlorocyclohexane ^b^	2.180	2.721	0.632		1.450			[25]
125	γ-Hexachlorocyclohexane ^b^	2.580	2.749	0.624		1.450			[25]
126	δ-Hexachlorocyclohexane	2.230	2.891	0.583		1.450			[25]
For the adsorption of polystyrene (PS) in seawater, Model (7)									
127	Pentachlorobenzene	5.280	4.830				1.138	5.220	[25]
128	Hexachlorobenzene ^b^	5.100	5.013				1.204	5.860	[25]
129	Phenanthrene	5.390	5.439				1.518	4.350	[25]
130	Fluoranthene	5.910	5.706				1.553	4.930	[25]
131	Anthracene	5.610	5.749				1.616	4.350	[25]
132	Pyrene	5.840	5.999				1.794	4.930	[25]
133	Chrysene ^b^	6.630	6.154				1.661	5.520	[25]
134	Benzoapyrene ^b^	6.920	6.740				1.924	6.110	[25]
135	Dibenzanthracene	7.520	6.826				1.847	6.700	[25]
136	Benzo[g,h,i]perylene	7.150	7.869				1.388	6.700	[25]
137	4-Fluorobenzoic acid	2.134	3.004				1.074	2.070	[14]
138	Trimethoprim ^b^	0.863	1.403				1.165	0.730	[26]
139	Sulfadiazine	0.833	0.708				1.174	−0.340	[26]
140	α-Hexachlorocyclohexane	3.190	2.849				1.024	4.260	[25]
141	β-Hexachlorocyclohexane	2.630	2.918				1.082	4.260	[25]
142	γ-Hexachlorocyclohexane	3.010	2.987				1.056	4.260	[25]
143	δ-Hexachlorocyclohexane	2.800	2.849				1.004	4.260	[25]
144	Perfluoropentanoic acid	2.412	1.774				0.701	2.810	[14]
145	Perfluorohexanoic acid ^b^	1.760	1.934				0.698	3.480	[14]
146	Perfluoroheptanoic acid	1.731	2.095				0.708	4.150	[14]
147	Perfluorodecanoic acid	2.669	2.550				0.755	6.150	[14]
148	Pentadecafluorooctanoic acid ^b^	3.220	2.229				0.723	4.810	[14]
149	Heptadecafluorooctanesulfonamide	2.792	2.217				0.789	5.800	[14]
150	Perfluoro-1-octanesulfonyl fluoride ^b^	2.147	3.618				0.721	7.840	[14]
151	Perfluoroundecanoic acid	2.752	2.710				0.748	6.820	[14]
152	Perfluorododecanoic acid	2.720	2.870				0.720	7.490	[14]
153	Pentacosafluorotridecanoic acid	3.162	3.031				0.741	8.160	[14]
154	Perfluorotetradecanoic acid ^b^	3.088	3.191				0.766	8.830	[14]

^a^ The unit of *K*_d_ is L/kg; ^b^ The compounds used for test subset in simulated external validation.

## Supplementary Materials

The following are available online, Models (S1)–(S10): Redeveloped models of the training and test subsets. Text S1: Computational details of the statistical parameters. Table S1: Coefficients, *VIF*, *t* and *p* values of the descriptors involved in log *K*_d_ models. Table S2: Molecular structures of all the studied organic compounds. Table S3: Experimental conditions of log *K*_d_ values and size of microplastics. Table S4: Parameter values for pp-LFERs. Table S5: Log *K*_ow_ values and quantum chemical descriptors. Figure S1: Williams plot for model (3). Figure S2: Fitting plots of experimental and predicted log *K*_d_ values by model (5). Figure S3: Distributions of prediction errors of log *K*_d_ calculated by model (5). Figure S4: Williams plot for the applicability domain of model (5). Figure S5: Fitting plots of experimental and predicted log *K*_d_ values by model (7). Figure S6: Distributions of prediction errors of log *K*_d_ calculated by model (7). Figure S7: Williams plot for the applicability domain by model (7).
