# Supplementary material for: Developing Predictive Models for Carrying Ability of Micro-Plastics towards Organic Pollutants
Source: Molecules. 2019 May 8;24(9):1784. doi: 10.3390/molecules24091784 (PMC6539320; doi:10.3390/molecules24091784)
Supplement: Supplementary file 1 [file molecules-24-01784-s001.pdf]

# Supporting Information

## Developing predictive models for carrying ability of micro-plastics towards organic pollutants

**Xiaoxuan Wei, Miao Li, Yifei Wang, Lingmin Jin, Guangcai Ma and Haiying Yu ***

College of Geography and Environmental Sciences, Zhejiang Normal University, Yingbin Avenue 688, 321004, Jinhua, P.R. China

* Correspondence: yhy@zjnu.cn; Tel.: 0086 579 8228 2273

Contents:

Models (S1)~(S10): Redeveloped models of the training and test subsets.

Text S1: Computational details of the statistical parameters.

Table S1: Coefficients, *VIF*, *t* and *p* values of the descriptors involved in log $K_d$ models.

Table S2: Molecular structures of all the studied organic compounds.

Table S3: Experimental conditions of log $K_d$ values and size of microplastics.

Table S4: Parameter values for pp-LFERs.

Table S5: Log $K_{OW}$ values and quantum chemical descriptors.

Figure S1: Williams plot for model (3).

Figure S2: Fitting plots of experimental and predicted log $K_d$ values by model (5).

Figure S3: Distributions of prediction errors of log $K_d$ calculated by model (5).

Figure S4: Williams plot for the applicability domain of model (5).

Figure S5: Fitting plots of experimental and predicted log $K_d$ values by model (7).

Figure S6: Distributions of prediction errors of log $K_d$ calculated by model (7).

Figure S7: Williams plot for the applicability domain by model (7).

The redeveloped models of the training sets are listed as following:

$$\log K_d = (-3.822 \pm 0.000) \times B + (3.054 \pm 0.000) \times V + (1.293 \pm 0.000) \times E + (-1.411 \pm 0.000) \quad \text{(S1)}$$

$$\log K_d = (-3.328 \pm 0.321) \times B + (6.046 \pm 1.299) \times V + (-4.807 \pm 2.482) \quad \text{(S2)}$$

$$\log K_d = (-2.517 \pm 0.122) \times B + (2.804 \pm 0.164) \times V + (1.085 \pm 0.259) \quad \text{(S3)}$$

$$\log K_d = (-3.248 \pm 0.242) \times B + (1.244 \pm 0.155) \times E + (3.225 \pm 0.353) \quad \text{(S4)}$$

$$\log K_d = (0.470 \pm 0.082) \times \log K_{OW} + (4.217 \pm 0.422) \times \pi + (-3.242 \pm 0.661) \quad \text{(S5)}$$

The regression models of the test sets are listed as following:

$$\log K_d = (-6.241 \pm 0.365) \times B + (2.901 \pm 0.356) \times V + (1.164 \pm 0.130) \times E + (-0.273 \pm 0.502) \quad \text{(S6)}$$

$$\log K_d = (-4.000 \pm 0.432) \times B + (2.729 \pm 1.419) \times V + (1.560 \pm 2.736) \quad \text{(S7)}$$

$$\log K_d = (-3.646 \pm 0.291) \times B + (3.088 \pm 0.083) \times V + (0.762 \pm 0.172) \quad \text{(S8)}$$

$$\log K_d = (-4.411 \pm 0.564) \times B + (1.399 \pm 0.200) \times E + (3.077 \pm 0.465) \quad \text{(S9)}$$

$$\log K_d = (0.392 \pm 0.164) \times \log K_{OW} + (4.006 \pm 0.872) \times \pi + (-2.838 \pm 1.415) \quad \text{(S10)}$$

**Text S1.** The computational formulations of the squared correlation coefficient $R^2$, predictive squared correlation coefficient $Q^2$, root-mean-square error (*RMSE*) and variance inflating factor (VIF) are shown here. The two statistics are used to quantify the validation and prediction performance of the developed QSPR models.

$$R^2 = 1 - \frac{\sum_{i=1}^{n}(y_i^{fit} - y_i^{exp})^2}{\sum_{i=1}^{n}(y_i^{exp} - \bar{y}^{exp})^2} \quad \text{(S11)}$$

$$Q^2 = 1 - \frac{\sum_{i=1}^{n}(y_i^{pred} - y_i^{exp})^2}{\sum_{i=1}^{n}(y_i^{exp} - \bar{y}^{exp})^2} \quad \text{(S12)}$$

$$RMSE = \sqrt{\frac{\sum_{i=1}^{n}(y_i^{pred} - y_i^{exp})^2}{n}} \quad \text{(S13)}$$

$$VIF = \frac{1}{1 - R_i^2} \quad \text{(S14)}$$

where, $y_i^{\text{fit}}$, $y_i^{\text{exp}}$, $\bar{y}^{\text{exp}}$, and $y_i^{\text{pred}}$ is the regression-fitted, experimental, average experimental, and predictive value of log$K_\text{d}$, respectively. $R_i^2$ is the determination coefficient for the regression of one parameter on all other independent variables in the dataset.

In statistics, the mean absolute error (*MAE*) is a quantity used to measure how close the predictive values are to the experimental values. The mean absolute error can be calculated by:

$$MAE = \frac{1}{n}\sum_{i=1}^{n}|E_i| , \quad |E_i| = |y_i^{a} - y_i^{b}| \quad \text{(S15)}$$

where, the $y_i^{\text{a}}$ is the predictive value and the $y_i^{\text{b}}$ is the experimental values.

A systematic error is an error that will occur consistently in only direction each time the experiment is performed and the values of the measurement will always be greater or lesser than the real values. Systematic errors most commonly arise from defects in the instrumentation or from using improper measuring techniques. The systematic error can be calculated by:

$$BIAS = \frac{1}{n}\sum_{i=1}^{n}E_i , \quad E_i = y_i^{a} - y_i^{b} \quad \text{(S15)}$$

where, the $y_i^{\text{a}}$ is the predictive value and the $y_i^{\text{b}}$ is the experimental values.

**Table S1.** Coefficients, *t* value of the *t* test, significance level (*p* value) and variance inflation factor (*VIF*) of the molecular structural descriptors involved in log $K_d$ models.

| Models | Parameters | Coefficients | *t* value | *p* value | *VIF* |
|---|---|---|---|---|---|
| Model (1) | *B* | −3.822 | −17.240 | <0.001 | 1.471 |
| | *V* | 3.054 | 8.776 | <0.001 | 1.464 |
| | *E* | 1.293 | 7.482 | <0.001 | 1.065 |
| Model (2) | *B* | −3.302 | −14.168 | <0.001 | 1.211 |
| | *V* | 5.594 | 6.307 | <0.001 | 1.211 |
| Model (4) | *B* | −2.594 | −25.788 | <0.001 | 1.395 |
| | *V* | 2.940 | 24.364 | <0.001 | 1.395 |
| Model (5) | *B* | −3.357 | −15.342 | <0.001 | 1.001 |
| | *E* | 1.299 | 10.229 | <0.001 | 1.001 |
| Model (6) | *A* | −14.645 | −6.945 | <0.001 | 1.000 |
| Model (7) | $\pi$ | 4.141 | 11.173 | <0.001 | 1.007 |
| | log$K_{ow}$ | 0.435 | 6.173 | <0.001 | 1.007 |

**Table S2.** Molecular structures and CAS number of all the studied organic compounds

| No. | Organic pollutants | CAS | Structures |
|---|---|---|---|
| 1 | 2,3-dichlorobiphenyl | 16605-91-7 | 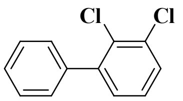 |
| 2 | 2,4'-dichlorobiphenyl | 34883-43-7 | 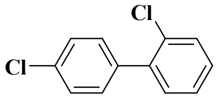 |
| 3 | 2,4,4'-trichlorobiphenyl | 7012-37-5 | 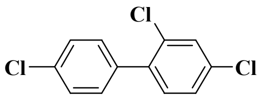 |
| 4 | 2,4',5-trichlorobiphenyl | 16606-02-3 | 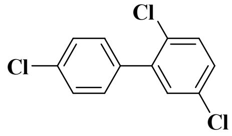 |

| No. | Organic pollutants | CAS | Structures |
|---|---|---|---|
| 5 | 2,2',5-trichlorobiphenyl | 37680-65-2 | 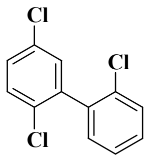 |
| 6 | 2,2',3,5'-tetrachlorobiphenyl | 41464-39-5 | 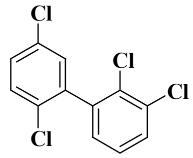 |
| 7 | 2,2',5,5'-tetrachlorobiphenyl | 35693-99-3 | 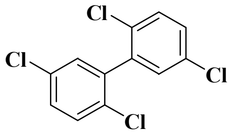 |
| 8 | 2,4,4',5-tetrachlorobiphenyl | 32690-93-0 | 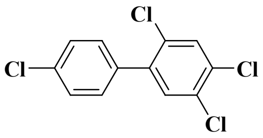 |
| 9 | 2,3',4,4'-tetrachlorobiphenyl | 32598-10-0 | 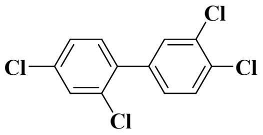 |
| 10 | 3,3',4,4'-tetrachlorobiphenyl | 32598-13-3 | 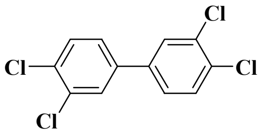 |
| 11 | 2,2',3,5-tetrachlorobiphenyl | 70362-46-8 | 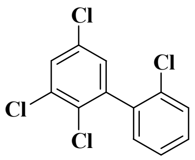 |
| 12 | 2,2',4,4'-tetrachlorobiphenyl | 2437-79-8 | 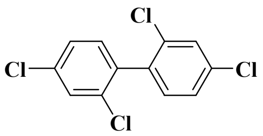 |
| 13 | 2,2',4,5,6'-pentachlorobiphenyl | 68194-06-9 | 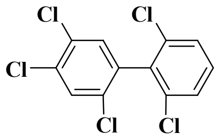 |
| 14 | 2,3,3',4,4'-pentachlorobiphenyl | 32598-14-4 | 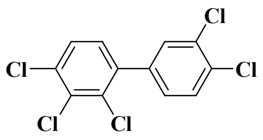 |

| No. | Organic pollutants | CAS | Structures |
|---|---|---|---|
| 15 | 2,3',4,4',5-pentachlorobiphenyl | 31508-00-6 | 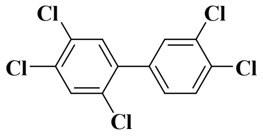 |
| 16 | 3,3',4,4',5-pentachlorobiphenyl | 57465-28-8 | 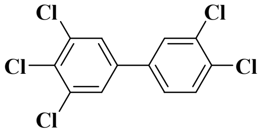 |
| 17 | 2,2',4,5,5'-pentachlorobiphenyl | 37680-73-2 | 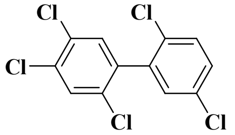 |
| 18 | 2,3,3',4',6-pentachlorobiphenyl | 38380-03-9 | 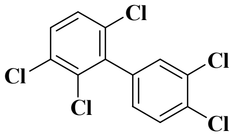 |
| 19 | 2,2',3,4',5-pentachlorobiphenyl | 68194-07-0 | 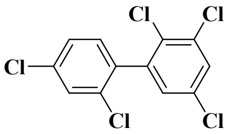 |
| 20 | 2,2',3,5',6-pentachlorobiphenyl | 38379-99-6 | 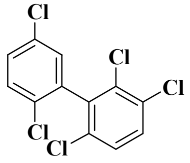 |
| 21 | 2,2',4,5',6-pentachlorobiphenyl | 60145-21-3 | 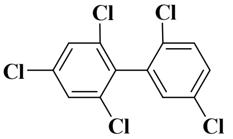 |
| 22 | 2,2',3,4',5,6-hexachlorobiphenyl | 68194-13-8 | 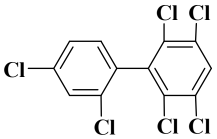 |
| 23 | 2,2',3,4,4',5'-hexachlorobiphenyl | 35065-28-2 | 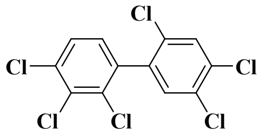 |
| 24 | 2,2',4,4',5,5'-hexachlorobiphenyl | 35065-27-1 | 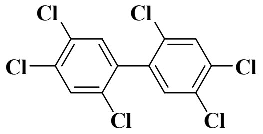 |

| No. | Organic pollutants | CAS | Structures |
|---|---|---|---|
| 25 | 2,3,3',4,4',5-hexachlorobiphenyl | 38380-08-4 | 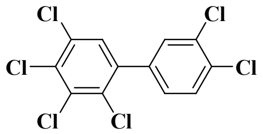 |
| 26 | 3,3',4,4',5,5'-hexachlorobiphenyl | 32774-16-6 | 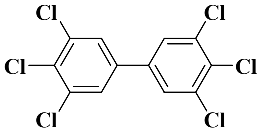 |
| 27 | 2,2',3,4,4',5-hexachlorobiphenyl | 35694-06-5 | 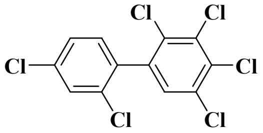 |
| 28 | 2,2',3,4',5',6-hexachlorobiphenyl | 38380-04-0 | 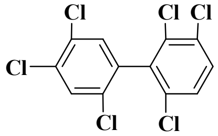 |
| 29 | 2,2',3,3',4,5-hexachlorobiphenyl | 55215-18-4 | 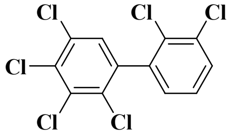 |
| 30 | 2,2',3,3',4,4'-hexachlorobiphenyl | 38380-07-3 | 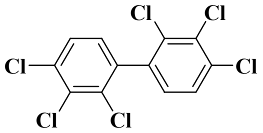 |
| 31 | 2,2',3,3',4,6'-hexachlorobiphenyl | 38380-05-1 | 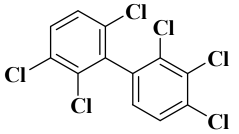 |
| 32 | 2,3,3',4,5,6-hexachlorobiphenyl | 41411-62-5 | 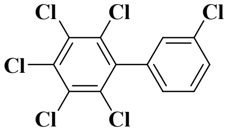 |
| 33 | 2,2',3,3',4,4',5-heptachlorobiphenyl | 35065-30-6 | 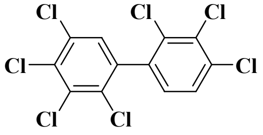 |
| 34 | 2,2',3,4,4',5,5'-heptachlorobiphenyl | 35065-29-3 | 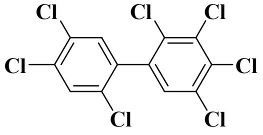 |

| No. | Organic pollutants | CAS | Structures |
|---|---|---|---|
| 35 | 2,2',3,4',5,5',6-heptachlorobiphenyl | 52663-68-0 | 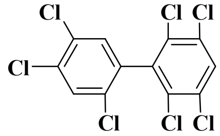 |
| 36 | Dichlorodiphenyltrichloroethane | 3547-04-4 | 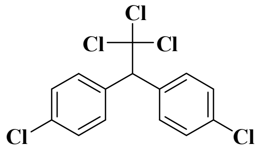 |
| 37 | Chlorobenzene | 108-90-7 | 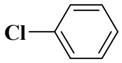 |
| 38 | Pentachlorobenzene | 608-93-5 | 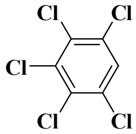 |
| 39 | Hexachlorobenzene | 118-74-1 | 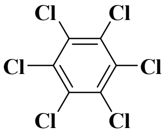 |
| 40 | Benzene | 71-43-2 | 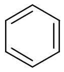 |
| 41 | Toluene | 108-88-3 | 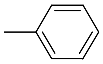 |
| 42 | Naphthalene | 91-20-3 | 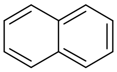 |
| 43 | 2-Methylanthracene | 613-12-7 | 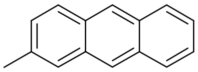 |
| 44 | 1-methylphenanthrene | 832-69-9 | 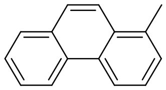 |
| 45 | 9,10-Dimethylanthracene | 781-43-1 | 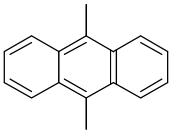 |
| 46 | 3,6-dimethylphenanthrene | 1576-67-6 | 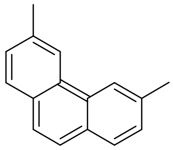 |
| 47 | Phenanthrene | 85-01-8 | 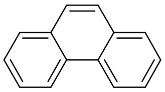 |
| 48 | Anthracene | 120-12-7 | 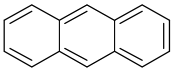 |

| No. | Organic pollutants | CAS | Structures |
|---|---|---|---|
| 49 | Pyrene | 129-00-0 | 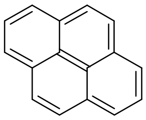 |
| 50 | Fluoranthene | 206-44-0 | 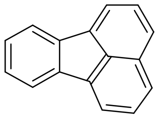 |
| 51 | Chrysene | 218-01-9 | 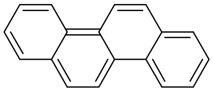 |
| 52 | Benzoapyrene | 50-32-8 | 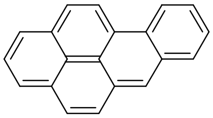 |
| 53 | Dibenzanthracene | 53-70-3 | 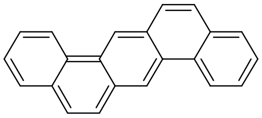 |
| 54 | Benzo[g,h,i]perylene | 191-24-2 | 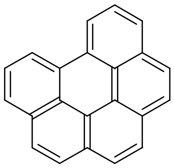 |
| 55 | 4-Fluorobenzoic acid | 456-22-4 | 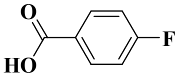 |
| 56 | Ethyl benzoate | 93-89-0 | 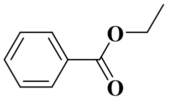 |
| 57 | Dioctyl phthalate | 117-81-7 | 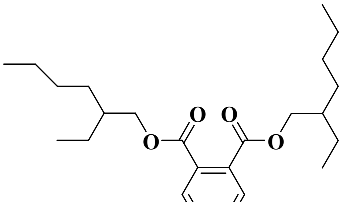 |
| 58 | Sulfadiazine | 68-35-9 | 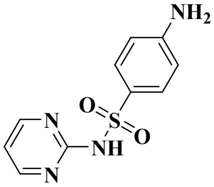 |

| No. | Organic pollutants | CAS | Structures |
|---|---|---|---|
| 59 | Trimethoprim | 738-70-5 | 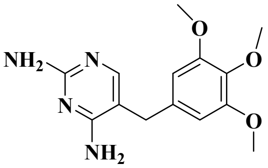 |
| 60 | Ciprofloxacin | 85721-33-1 | 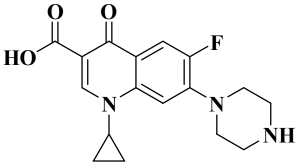 |
| 61 | Oxytetracycline | 79-57-2 | 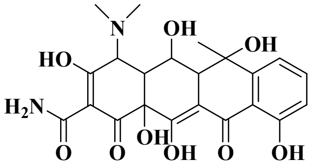 |
| 62 | Cyclohexane | 110-82-7 | 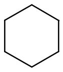 |
| 63 | α-Hexachlorocyclohexane | 319-84-6 | 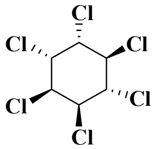 |
| 64 | β-Hexachlorocyclohexane | 319-85-7 | 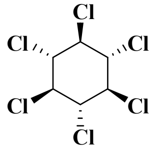 |
| 65 | γ-Hexachlorocyclohexane | 58-89-9 | 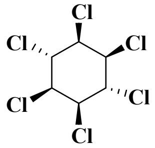 |
| 66 | δ-Hexachlorocyclohexane | 319-86-8 | 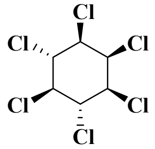 |
| 67 | Hexane | 110-54-3 | 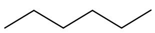 |
| 68 | Perfluoropentanoic acid | 2706-90-3 | $F_3C-[CF_2]_3-COOH$ |
| 69 | Perfluorohexanoic acid | 307-24-4 | $F_3C-[CF_2]_4-COOH$ |

| No. | Organic pollutants | CAS | Structures |
|---|---|---|---|
| 70 | Perfluoroheptanoic acid | 375-85-9 | $F_3C-[CF_2]_5-COOH$ |
| 71 | Pentadecafluorooctanoic acid | 335-67-1 | $F_3C-[CF_2]_6-COOH$ |
| 72 | Heptadecafluorooctanesulfonamide | 754-91-6 | $F_3C-[CF_2]_7-SO_2NH_2$ |
| 73 | Perfluoro-1-octanesulfonyl fluoride | 307-35-7 | $F_3C-[CF_2]_7-SFO_2$ |
| 74 | Perfluorodecanoic acid | 335-76-2 | $F_3C-[CF_2]_8-COOH$ |
| 75 | Perfluoroundecanoic acid | 2058-94-8 | $F_3C-[CF_2]_9-COOH$ |
| 76 | Perfluorododecanoic acid | 307-55-1 | $F_3C-[CF_2]_{10}-COOH$ |
| 77 | Pentacosafluorotridecanoic acid | 72629-94-8 | $F_3C-[CF_2]_{11}-COOH$ |
| 78 | Perfluorotetradecanoic acid | 376-06-7 | $F_3C-[CF_2]_{12}-COOH$ |

**Table S3.** Experimental conditions of log $K_d$ values and size of microplastics

| Microplastic | Water type | Temperature range (°C) | Particle size range (μm) | Ref |
|---|---|---|---|---|
| PE | Seawater | 18~25 | 10-440 | [1-5] |
| PE | Freshwater | 18~25 | 10-180 | [1,5,6] |
| PE | Pure water | 25 | 20-250 | [2,7-9] |
| PP | Seawater | 12~25 | 75-180, | [1] |
| | | | 320-440, | [3,10] |
| | | | 1000-5000 | [10] |
| PS | Seawater | 25 | 320-440 | [1,3,11] |

**Table S4.** Parameter values for pp-LFERs

| No. | Organic compounds | *E* | *S* | *A* | *B* | *V* |
|---|---|---|---|---|---|---|
| 1 | 2,3-dichlorobiphenyl | 1.628 | 1.198 | 0.000 | 0.163 | 1.547 |
| 2 | 2,4'-dichlorobiphenyl | 1.620 | 1.198 | 0.000 | 0.166 | 1.552 |
| 3 | 2,4,4'-trichlorobiphenyl | 1.758 | 1.310 | 0.000 | 0.129 | 1.670 |
| 4 | 2,4',5-trichlorobiphenyl | 1.766 | 1.334 | 0.000 | 0.132 | 1.674 |
| 5 | 2,2',5-trichlorobiphenyl | 1.758 | 1.310 | 0.000 | 0.145 | 1.648 |
| 6 | 2,2',3,5'-tetrachlorobiphenyl | 1.905 | 1.443 | 0.000 | 0.150 | 1.770 |
| 7 | 2,2',5,5'-tetrachlorobiphenyl | 1.903 | 1.423 | 0.000 | 0.147 | 1.770 |
| 8 | 2,4,4',5-tetrachlorobiphenyl | 1.903 | 1.473 | 0.000 | 0.130 | 1.792 |
| 9 | 2,3',4,4'-tetrachlorobiphenyl | 1.903 | 1.473 | 0.000 | 0.130 | 1.792 |
| 10 | 3,3',4,4'-tetrachlorobiphenyl | 1.915 | 1.503 | 0.000 | 0.110 | 1.814 |
| 11 | 2,2',3,5-tetrachlorobiphenyl | 1.905 | 1.443 | 0.000 | 0.150 | 1.770 |
| 12 | 2,2',4,4'-tetrachlorobiphenyl | 1.890 | 1.443 | 0.000 | 0.150 | 1.770 |
| 13 | 2,2',3,4',5-pentachlorobiphenyl | 2.045 | 1.575 | 0.000 | 0.130 | 1.893 |
| 14 | 2,2',3,5',6-pentachlorobiphenyl | 2.045 | 1.545 | 0.000 | 0.130 | 1.871 |
| 15 | 2,2',4,5',6-pentachlorobiphenyl | 2.038 | 1.545 | 0.000 | 0.130 | 1.871 |
| 16 | 2,2',4,5,6'-pentachlorobiphenyl | 2.038 | 1.545 | 0.000 | 0.130 | 1.871 |
| 17 | 2,3,3',4,4'-pentachlorobiphenyl | 2.035 | 1.603 | 0.000 | 0.110 | 1.922 |
| 18 | 2,3',4,4',5-pentachlorobiphenyl | 2.050 | 1.606 | 0.000 | 0.110 | 1.919 |
| 19 | 3,3',4,4',5-pentachlorobiphenyl | 2.075 | 1.643 | 0.000 | 0.090 | 1.936 |
| 20 | 2,2',4,5,5'-pentachlorobiphenyl | 2.043 | 1.530 | 0.000 | 0.133 | 1.893 |
| 21 | 2,3,3',4',6-pentachlorobiphenyl | 2.045 | 1.575 | 0.000 | 0.130 | 1.893 |
| 22 | 2,2',3,4',5,6-hexachlorobiphenyl | 2.188 | 1.675 | 0.000 | 0.110 | 1.993 |

| No. | Organic compounds | *E* | *S* | *A* | *B* | *V* |
|---|---|---|---|---|---|---|
| 23 | 2,2',4,4',5,5'-hexachlorobiphenyl | 2.183 | 1.473 | 0.000 | 0.113 | 2.015 |
| 24 | 2,3,3',4,4',5-hexachlorobiphenyl | 2.196 | 1.742 | 0.000 | 0.090 | 2.041 |
| 25 | 3,3',4,4',5,5'-hexachlorobiphenyl | 2.183 | 1.783 | 0.000 | 0.070 | 2.059 |
| 26 | 2,2',3,4,4',5-hexachlorobiphenyl | 2.185 | 1.708 | 0.000 | 0.110 | 2.015 |
| 27 | 2,2',3,3',4,5-hexachlorobiphenyl | 2.193 | 1.708 | 0.000 | 0.110 | 2.015 |
| 28 | 2,2',3,4,4',5'-hexachlorobiphenyl | 2.183 | 1.718 | 0.000 | 0.110 | 2.009 |
| 29 | 2,2',3,4',5',6-hexachlorobiphenyl | 2.188 | 1.675 | 0.000 | 0.110 | 1.993 |
| 30 | 2,2',3,3',4,4'-hexachlorobiphenyl | 2.185 | 1.708 | 0.000 | 0.110 | 2.015 |
| 31 | 2,2',3,3',4,6'-hexachlorobiphenyl | 2.188 | 1.675 | 0.000 | 0.110 | 1.993 |
| 32 | 2,3,3',4,5,6-hexachlorobiphenyl | 2.193 | 1.708 | 0.000 | 0.110 | 2.015 |
| 33 | 2,2',3,3',4,4',5-heptachlorobiphenyl | 2.333 | 1.840 | 0.000 | 0.090 | 2.138 |
| 34 | 2,2',3,4,4',5,5'-heptachlorobiphenyl | 2.298 | 1.850 | 0.000 | 0.090 | 2.131 |
| 35 | 2,2',3,4',5,5',6-heptachlorobiphenyl | 2.338 | 1.805 | 0.000 | 0.090 | 2.116 |
| 36 | Dichlorodiphenyltrichloroethane | 1.810 | 1.765 | 0.000 | 0.180 | 2.218 |
| 37 | Chlorobenzene | 0.720 | 0.650 | 0.000 | 0.070 | 0.839 |
| 38 | Pentachlorobenzene | 1.330 | 0.944 | 0.060 | 0.000 | 1.328 |
| 39 | Hexachlorobenzene | 1.475 | 0.939 | 0.000 | 0.130 | 1.451 |
| 40 | Benzene | 0.610 | 0.510 | 0.000 | 0.140 | 0.716 |
| 41 | Toluene | 0.600 | 0.520 | 0.000 | 0.140 | 0.857 |
| 42 | Naphthalene | 1.340 | 0.919 | 0.000 | 0.199 | 1.085 |
| 43 | 2-Methylanthracene | 2.290 | 1.300 | 0.000 | 0.310 | 1.595 |
| 44 | 1-methylphenanthrene | 2.060 | 1.250 | 0.000 | 0.275 | 1.595 |
| 45 | 9,10-Dimethylanthracene | 2.236 | 1.270 | 0.000 | 0.300 | 1.736 |

| No. | Organic compounds | ***E*** | ***S*** | ***A*** | ***B*** | ***V*** |
|---|---|---|---|---|---|---|
| 46 | 3,6-dimethylphenanthrene | 2.053 | 1.290 | 0.000 | 0.290 | 1.736 |
| 47 | Phenanthrene | 2.033 | 1.292 | 0.000 | 0.276 | 1.454 |
| 48 | Anthracene | 2.077 | 1.329 | 0.000 | 0.272 | 1.454 |
| 49 | Pyrene | 2.698 | 1.669 | 0.000 | 0.282 | 1.585 |
| 50 | Fluoranthene | 2.354 | 1.527 | 0.000 | 0.247 | 1.585 |
| 51 | Chrysene | 2.897 | 1.709 | 0.000 | 0.325 | 1.823 |
| 52 | Benzoapyrene | 3.554 | 1.960 | 0.000 | 0.417 | 1.954 |
| 53 | Dibenzanthracene | 3.972 | 2.058 | 0.000 | 0.462 | 2.192 |
| 54 | Benzo[g,h,i]perylene | 4.004 | 1.930 | 0.000 | 0.455 | 2.084 |
| 55 | Ethyl benzoate | 0.690 | 0.853 | 0.000 | 0.459 | 1.214 |
| 56 | Dioctyl phthalate | 0.650 | 1.277 | 0.000 | 1.088 | 3.401 |
| 57 | Sulfadiazine | 2.080 | 2.550 | 0.650 | 1.370 | 1.723 |
| 58 | Trimethoprim | 1.962 | 2.382 | 0.207 | 1.832 | 2.181 |
| 59 | Ciprofloxacin | 2.200 | 2.340 | 0.700 | 2.520 | 2.305 |
| 60 | Oxytetracycline | 3.600 | 3.050 | 1.650 | 3.500 | 3.158 |
| 61 | Cyclohexane | 0.310 | 0.113 | 0.000 | 0.000 | |
| 62 | α-Hexachlorocyclohexane | 1.450 | 0.940 | 0.150 | 0.620 | 1.580 |
| 63 | β-Hexachlorocyclohexane | 1.450 | 1.068 | 0.260 | 0.632 | 1.580 |
| 64 | γ-Hexachlorocyclohexane | 1.450 | 1.026 | 0.170 | 0.624 | 1.580 |
| 65 | δ-Hexachlorocyclohexane | 1.450 | 1.108 | 0.270 | 0.583 | 1.580 |
| 66 | Hexane | 0.000 | 0.025 | 0.000 | 0.000 | 0.954 |

**Table S5.** Log $K_{OW}$ values and quantum chemical descriptors

| No | Organic compounds | log $K_{ow}$ | $M_w$ a.u. | $\varepsilon_\alpha$ eV | $\varepsilon_\beta$ eV | $qH^+$ acu | $q^-$ acu | $v'$ $cm^3/mol$ | $\pi$ |
|---|---|---|---|---|---|---|---|---|---|
| 1 | Pentachlorobenzene | 5.220 | 247.852 | 0.246 | 0.339 | 0.168 | -0.081 | 133.966 | 1.138 |
| 2 | Hexachlorobenzene | 5.860 | 281.813 | 0.234 | 0.344 | - | -0.067 | 141.531 | 1.204 |
| 3 | Phenanthrene | 4.350 | 178.078 | 0.254 | 0.294 | 0.105 | -0.157 | 136.263 | 1.518 |
| 4 | Anthracene | 4.350 | 178.078 | 0.230 | 0.276 | 0.105 | -0.221 | 137.049 | 1.616 |
| 5 | Perylene | 4.930 | 202.078 | 0.236 | 0.279 | 0.106 | -0.174 | 141.302 | 1.794 |
| 6 | Fluoranthene | 4.930 | 202.078 | 0.226 | 0.296 | 0.111 | -0.168 | 156.458 | 1.553 |
| 7 | Chrysene | 5.520 | 228.094 | 0.243 | 0.287 | 0.106 | -0.160 | 171.341 | 1.661 |
| 8 | Benzoapyrene | 6.110 | 252.094 | 0.226 | 0.271 | 0.107 | -0.253 | 180.919 | 1.924 |
| 9 | Dibenzanthracene | 6.700 | 278.110 | 0.235 | 0.283 | 0.108 | -0.238 | 194.648 | 1.847 |
| 10 | Benzo[g,h,i]perylene | 6.700 | 230.110 | 0.230 | 0.246 | 0.135 | -0.275 | 182.472 | 1.388 |
| 11 | 4-Fluorobenzoic acid | 2.070 | 140.027 | 0.245 | 0.339 | 0.350 | -0.504 | 88.566 | 1.074 |
| 12 | Sulfadiazine | -0.340 | 250.052 | 0.249 | 0.297 | 0.322 | -0.684 | 169.805 | 1.174 |
| 13 | Trimethoprim | 0.730 | 290.138 | 0.281 | 0.291 | 0.286 | -0.651 | 209.410 | 1.165 |
| 14 | α-Hexachlorocyclohexane | 4.260 | 287.860 | 0.254 | 0.386 | 0.225 | -0.043 | 152.922 | 1.024 |
| 15 | β-Hexachlorocyclohexane | 4.260 | 287.860 | 0.237 | 0.382 | 0.216 | -0.182 | 145.969 | 1.082 |
| 16 | γ-Hexachlorocyclohexane | 4.260 | 287.860 | 0.257 | 0.378 | 0.225 | -0.202 | 147.124 | 1.056 |
| 17 | δ-Hexachlorocyclohexane | 4.260 | 287.860 | 0.244 | 0.386 | 0.223 | -0.197 | 155.934 | 1.004 |
| 18 | Perfluoropentanoic acid | 2.810 | 263.983 | 0.252 | 0.387 | 0.371 | -0.467 | 100.143 | 0.701 |
| 19 | Perfluorohexanoic acid | 3.480 | 313.980 | 0.251 | 0.387 | 0.371 | -0.467 | 119.440 | 0.698 |
| 20 | Perfluoroheptanoic acid | 4.150 | 363.977 | 0.251 | 0.387 | 0.371 | -0.467 | 136.286 | 0.708 |
| 21 | Pentadecafluorooctanoic acid | 4.810 | 413.974 | 0.250 | 0.388 | 0.371 | -0.455 | 151.904 | 0.723 |
| 22 | Heptadecafluorooctanesulfonamide | 5.800 | 498.953 | 0.277 | 0.397 | 0.341 | -0.694 | 180.121 | 0.789 |

| No | Organic compounds | log $K_{ow}$ | $M_w$ a.u. | $\varepsilon_\alpha$ eV | $\varepsilon_\beta$ eV | $q\text{H}^+$ acu | $q^-$ acu | $v'$ cm$^3$/mol | $\pi$ |
|---|---|---|---|---|---|---|---|---|---|
| 23 | Perfluoro-1-octanesulfonyl fluoride | 7.840 | 501.933 | 0.255 | 0.417 | - | -0.457 | 186.953 | 0.721 |
| 24 | Perfluorodecanoic acid | 6.150 | 513.967 | 0.250 | 0.388 | 0.371 | -0.456 | 180.358 | 0.755 |
| 25 | Perfluoroundecanoic acid | 6.820 | 563.964 | 0.251 | 0.388 | 0.371 | -0.467 | 199.076 | 0.748 |
| 26 | Perfluorododecanoic acid | 7.490 | 613.961 | 0.251 | 0.388 | 0.371 | -0.462 | 226.064 | 0.720 |
| 27 | Pentacosafluorotridecanoic acid | 8.160 | 663.958 | 0.251 | 0.388 | 0.371 | -0.455 | 236.926 | 0.741 |
| 28 | Perfluorotetradecanoic acid | 8.830 | 713.955 | 0.251 | 0.388 | 0.371 | -0.455 | 246.230 | 0.766 |

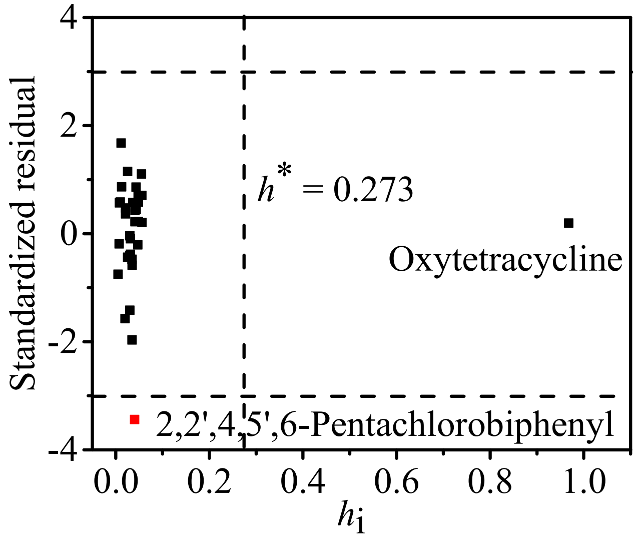


**Figure S1.** Williams plot for model (3)

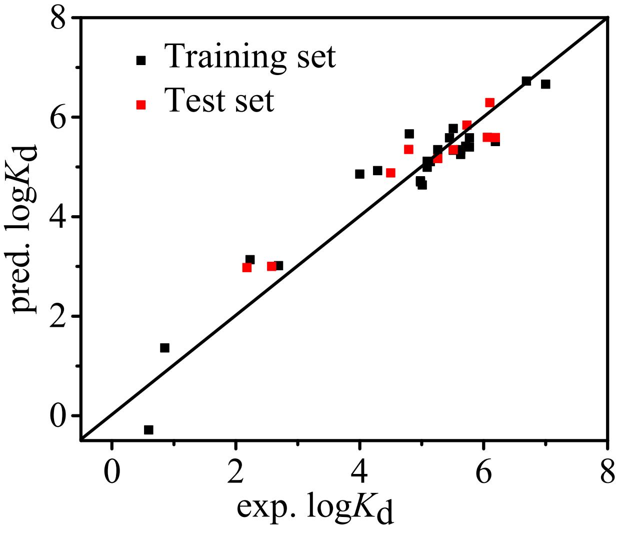


**Figure S2.** Fitting plots of experimental and predicted log $K_d$ values by model (5).

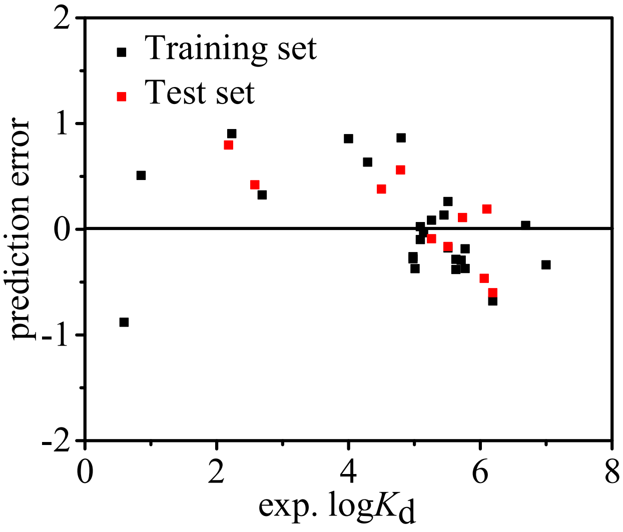


**Figure S3.** Distributions of prediction errors of log $K_d$ calculated by model (5).

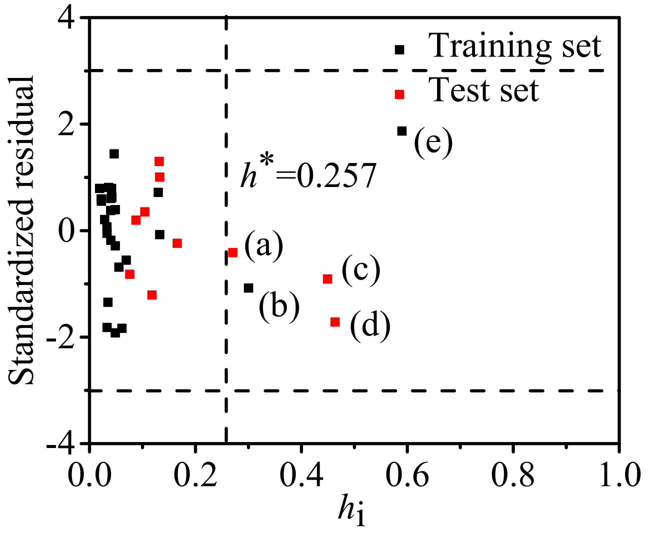


**Figure S4**. Williams plot for the applicability domain of model (5). The $h_i$ refers to the verse leverage value. (a): benzoapyrene, (b): sulfadiazine, (c): γ-hexachlorocyclohexane, (d): β-hexachlorocyclohexane, (e): trimethoprim.

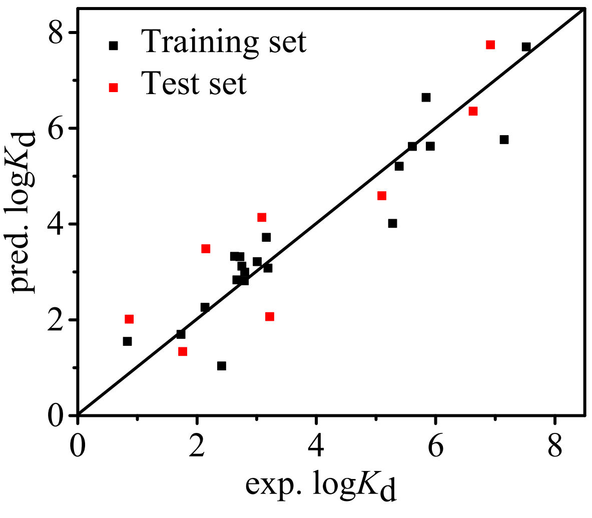


**Figure S5.** Fitting plots of experimental and predicted log $K_d$ values by model (7).

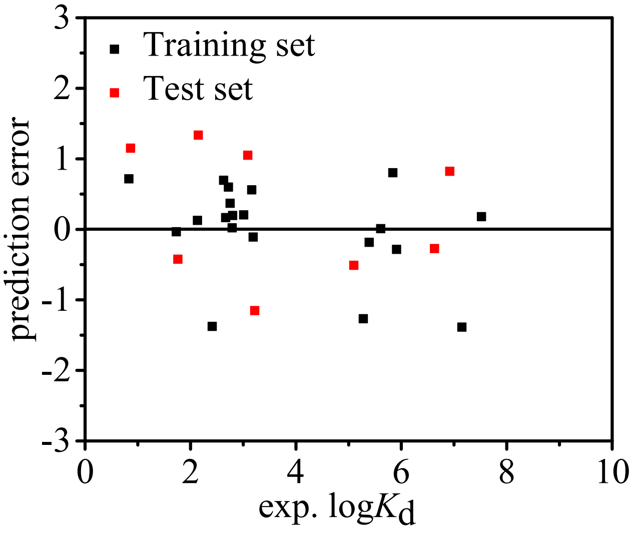


**Figure S6.** Distributions of prediction errors of log $K_d$ calculated by model (7).

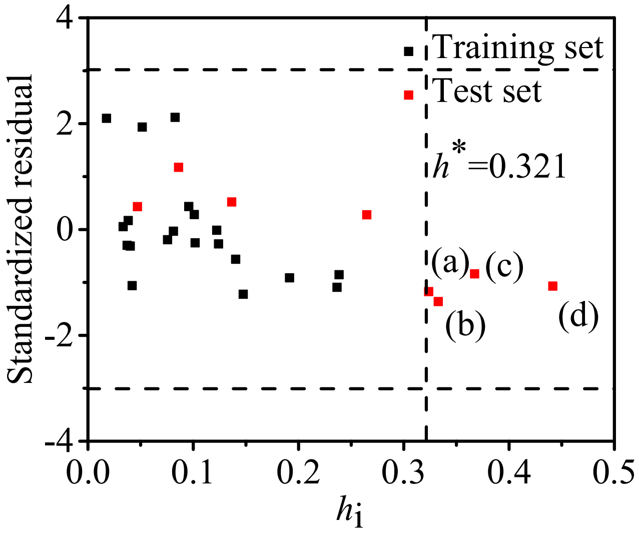


**Figure S7.** Williams plot for the applicability domain by model (7). Williams plot for the applicability domain of model (5). The $h_i$ refers to the verse leverage value. (a): trimethoprim, (b): perfluoro-1-octanesulfonyl fluoride, (c): benzoapyrene, (d): perfluorotetradecanoic acid.

## References:


1. Li, J.; Zhang, K.; Zhang, H. Adsorption of antibiotics on microplastics. *Environ. Pollut.* **2018**, *237*,460-467.
2. Zhang, K.N.; Li, J.; Li, X.Q.; Zhang, H. Mechanisms and kinetics of oxytetracycline adsorption-desorption onto microplastics. *Environ. Chem.* **2017**, *36*, 2531-2540.
3. Hwang, L.; Won Joon, S.; Jung-Hwan, K. Sorption capacity of plastic debris for

hydrophobic organic chemicals. *Sci. Total Environ.* **2014**, *470-471*, 1545-1552.

4. Bakir, A.; Rowland, S.J.; Thompson, R.C. Enhanced desorption of persistent organic pollutants from microplastics under simulated physiological conditions. *Environ. Pollut.* **2014**, *185*, 16-23.
5. Velzeboer, I.; Kwadijk, C.J.A.F; Koelmans, A.A. Strong sorption of PCBs to nanoplastics, microplastics, carbon nanotubes, and fullerenes. *Environ. Sci. Technol.* **2014**, *48*, 4869-4876.
6. Teuten, E.L.; Rowland, S.J.; Galloway, T.S. Potential for plastics to transport hydrophobic contaminants. *Environ. Sci. Technol*. **2007**, *41*, 7759-7764.
7. Fernandez, L.A.; Macfarlane, J.K.; Tcaciuc, A.P. Measurement of freely dissolved PAH concentrations in sediment beds using passive sampling with low-density polyethylene strips. *Environ. Sci. Technol.* **2009**, *43*, 1430-1436.
8. Huffer, T.; Hofmann, T. Sorption of non-polar organic compounds by micro-sized plastic particles in aqueous solution. *Environ. Pollut.* **2016**, *214*, 194-201.
9. Pascall, M.A.; Zabik, M.E.; Zabik, M.J. Uptake of polychlorinated biphenyls (PCBs) from an aqueous medium by polyethylene, polyvinyl chloride, and polystyrene films. *J. Agr. Food Chem.* **2005**, *53*,164-169.
10. Mato, Y.; Isobe, T.; Takada, H. Plastic resin pellets as a transport medium for toxic chemicals in the marine environment. *Environ. Sci. Technol.* **2001**, *35*, 318-324.
11. Llorca, M.; Schirinzi, G.; Martínez, M.; Barceló, D.; Farréa, M. Adsorption of perfluoroalkyl substances on microplastics under environmental conditions. *Environ. Pollut.* **2018**, *235*,680.
